# Fermented Broth in Tyrosinase- and Melanogenesis Inhibition

**DOI:** 10.3390/molecules190913122

**Published:** 2014-08-26

**Authors:** Chin-Feng Chan, Ching-Cheng Huang, Ming-Yuan Lee, Yung-Sheng Lin

**Affiliations:** 1 Department of Applied Cosmetology and Master Program of Cosmetic Science, Hungkuang University, Taichung 43302, Taiwan; E-Mails: cfchan@sunrise.hk.edu.tw (C.-F.C.); junas.tw@yahoo.com.tw (C.-C.H.); mingyuan761016@gmail.com (M.-Y.L.); 2 Department of Biomedical Engineering, Mingchuan University, Gui Shan District, Taoyuan 333, Taiwan

**Keywords:** microorganisms, fermented broth, melanogenesis

## Abstract

Fermented broth has a long history of applications in the food, pharmaceutical and cosmetic industries. Recently, the use of fermented broth in skin care products is in ascendance. This review investigates the efficacy of fermented broth in inhibiting tyrosinase and melanogenesis. Possible active ingredients and hypopigmentation mechanisms of fermented broth are discussed, and potential applications of fermented broth in the cosmetic industry are also addressed.

## 1. Introduction

In the global beauty industry, multinational corporations such as L’Oreal, Estée Lauder, and Procter & Gamble earn billions of dollars every year by selling skin care products and cosmetics [1]. The ideal skin tone, according to traditional Eastern Asian cultural beliefs, is white [2]. Since the Edo period in Japan (1603–1868), women have had a “moral duty” to apply white powder to their faces [3]. In South Korea, both women and men strive for skin that is as white as pale jade [2]. As the old Chinese adage states, “A white complexion is powerful enough to hide seven faults” [4]. For these reasons, Eastern Asian women have sought pale white skin for hundreds of years [5]. A study of the sexual preference of women and men in China determined that the male participants preferred women with lighter skin tones over those with darker complexions [6]. Another study indicated that tan skin indicates a blue collar socioeconomic status, whereas pale skin represents a white collar status [1]. Therefore, numerous skin care products labeled skin “whitening” or “lightening” comprise the best-selling product lines in the Asian cosmetics market [7]. Scientists have proved that numerous skin-whitening agents added to products effectively reduce melanin, which is the main source of skin color [8].

When humans are exposed to unlimited UV light, melanogenesis causes hyperpigmentation through the overproduction of melanin, which causes the skin to tan [9]. Tyrosinase is the key enzyme in the melanin biosynthesis pathway [10]. Hydroxylated l-tyrosine produces l-3,4-dihydroxyphenylalanine (l-DOPA), which is oxidized to the corresponding *o*-quinone [11]. Tyrosinase inhibitors can be categorized into competitive, uncompetitive, and noncompetitive inhibitors [12]. Although most methods for skin lightening involve inhibiting tyrosinase [13], skin lightening can be achieved using other methods, such as epidermal turnover enhancement [14,15,16,17] and antioxidation agents [18]. Enhancers of epidermal turnover reduce melanin by accelerating epidermal turnover time [19,20], and antioxidants reduce reactive oxygen species that induce melanogenesis caused by UV irradiation [21].

Tyrosinase inhibitors such as l-ascorbic acid [22,23,24], kojic acid [25,26,27], ellagic acid [28,29,30], tranexamic acid [31,32,33], and hydroquinone [34,35,36] have been used as skin-whitening agents, albeit not without problems. For example, l-ascorbic acid is heat sensitive and degrades easily [37]; kojic acid can trigger allergic reactions such as contact dermatitis and sensitization [38] and has carcinogenic potential [39,40]; ellagic acid is insoluble and has poor bioavailability [41]; the melanogenesis inhibition pathway of tranexamic acid remains undetermined [32]; and long-term use of hydroquinone causes exogenous ochronosis [42,43]. Epidermal turnover enhancers only remove the melanin in the uppermost layer of the epidermis [44], and antioxidants, such as various botanical extracts, induce contact dermatitis [45]. Because of these problems, scientists have endeavored to develop alternative skin-whitening techniques.

**Figure 1:**
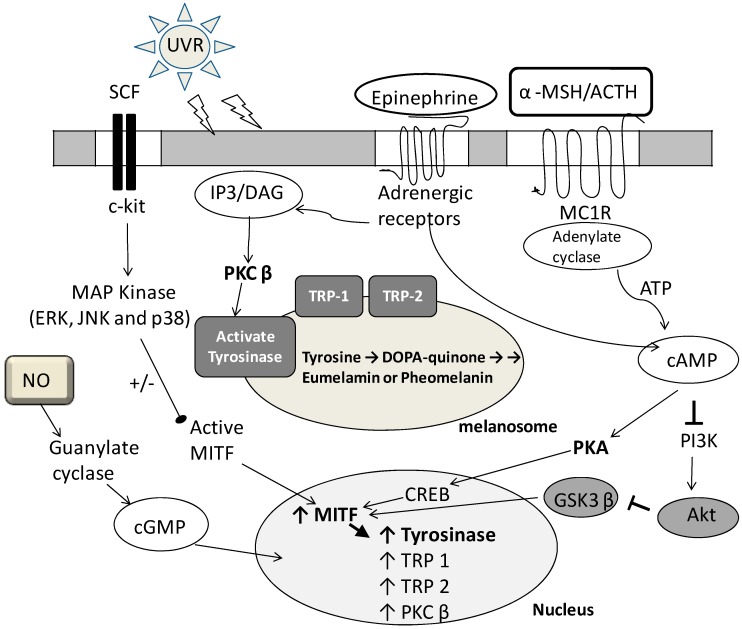
Scheme presentation of different signal pathways to regulate melanogenesis. A lot of receptor activation factors, second messengers, and melanogenic enzymes are involved in melanin synthesis (adapted from [53,54,55,56]).

Mushroom tyrosinase has been used for prescreening hypopigmentation agents because it is commercially available. However, it was found that many melanogenesis inhibitors didn’t exhibit inhibitory effects on mushroom tyrosinase activity [46,47,48,49]. Mushroom tyrosinase is a secreted form which doesn’t resemble mammalian tyrosinase [50,51,52]. Cytosolic mammalian tyrosinase is an inactive form which will be glycosylated in the Golgi complex. Then, glycosylated mammalian tyrosinase is delivered to melanosomes wherein the enzyme is membrane-bound form and phosphoactivated by PKCβ [50,53,54]. In addition, melanogenesis involves sophisticated signal pathways (Figure 1) [53,54,55,56]. For example, UV radiation (UVR), the most important extrinsic factor of melanogenesis, increases α-melanocyte-stimulating hormone (α-MSH) binding to melanocortin 1 receptor (MC1R) followed by activating adenylate cyclase, increasing cAMP levels, activating the enzyme protein kinase A (PKA), and inducing gene transcription of microphthalmia-associated transcription factor (MITF), leading to transcription of the melanogenic enzyme tyrosinase [53,54,57,58,59,60,61]. Norepinephrine/α1 or β2 adrenergic receptor and UV radiation induce diacylglycerol (DAG) release from the cell membrane. DAG activates protein kinase C-β (PKC-β), which then phosphorylates serine residues on tyrosinase and activates the enzyme [53,54]. Binding stem cell factor (SCF) to c-kit results in dimerization of receptors followed by activating mitogen-activated protein kinase (MAPK) cascade [53,62]. The MAPK family proteins, including p38, ERK, and JNK, are known to play crucial roles in melanogenesis [56,62,63]. The ERK and JNK pathways cause downregulation (−) of melanin synthesis. In contrast, the phosphorylation of p38 will activate (+) MITF expression, which in turn transcriptionally upregulates the expression of melanogenic enzymes such as tyrosinase, TRP-1, and TRP-2, eventually inducing melanin production [55,56,57] (Figure 1). The nitric oxide (NO) signaling pathway plays a very important role in UVR-induced melanogenesis. Enhancement of tyrosinase gene expression via the cGMP pathway with the expression of MITF is a primary mechanism of NO-induced melanogenesis [53,54,64]. Inhibition of phosphatidylinositol 3-kinase (PI3K) by cAMP will result in reduction of AKT phosphorylation and its activation. Therefore, AKT cannot phosphorylate GSK 3β. In turn cAMP decreases the phosphorylation of GSK 3β and stimulates its activity [55,62,65,66] (Figure 1). Therefore, activation of GSK3β by cAMP facilitates MITF binding to the tyrosinase promoter and leading to stimulation of melanogenesis. The hypopigmentation mechanism of many melanogenesis inhibitors is to modulate the regulators of signal pathways instead of inhibiting tyrosinase activity. Therefore, the mushroom tyrosinase inhibitors have to be proved to inhibit melanogenesis *in vitro*, or they are not classified to hypopigmentation agents. This study investigates the difference between tyrosinase inhibitors and melanogenesis inhibitors of fermented broths including active ingredients, signal pathway regulators and mechanism.

Microorganisms such as bacteria, yeasts, and molds are commonly employed in fermentation [67]. For example, probiotics are used in dairy products, fermented vegetables, fish and sausages, and as silage inoculants [68]. Human clinical trials have explored the use of numerous probiotic supplements to treat skin disorders [69]. Skin products containing fermented broth have been launched in the market. Pitera by SK-II is fermented by yeast to whiten the skin [70]. Numerous reports have indicated that fermented broth can inhibit tyrosinase activity and melanogenesis [9,11,46,47,48,49,71]. However, no study has reviewed these findings, thus this review describes the process by which fermented broth suppresses melanogenesis and the possible active whitening ingredients found in the broth.

## 2. Fermented Broth in Inhibiting Melanogenesis

### 2.1. Fermentation Process

Fermentation is a metabolic process in which sugars are converted into acids, gases, and alcohol [72], mainly with the involvement of yeasts, bacteria, and fungi. The fermented broth contains complex products [73]. Applications for fermented broth include biotherapeutics, biological materials, and ethanol production [73]. In addition, fermented broth has recently been used to reduce melanin hyperpigmentation. Table 1 summarizes applications of fermented broths in reduction of mushroom tyrosinase activity and melanogenesis in B16 cells. Specific materials and conditions required for fermentation include broth, microorganisms, a suitable temperature, and appropriate fermentation time.

Eight of twenty five fermented broths were proved to be tyrosinase and melanogenesis inhibitors [9,11,74,75,76,77,78,79]. Twelve fermented broths were proved to be tyrosinase inhibitors, but not melanogenesis inhibitors [71,80,81,82,83,84,85,86,87,88]. Four fermented broths showed melanogenesis inhibition but no inhibition of tyrosinase activity [46,47,48,49]. It is worth mentioning that both 8'-hydroxydaidzein (8'-ODI) and 3'-hydroxydaidzein (3'-ODI) can be obtained from soybean/*Aspergillus oryzae* fermented broth [47] but while 8'-ODI exhibited inhibitory effect on mushroom tyrosinse activity, it did not exhibit intracellular tyrosinse activity in B16F10 cells [47], 3'-ODI had no effects on either mushroom tyrosinse or intracellular tyrosinse activity [47], indicating that the hypopigmentation mechanism of melanogenesis inhibitors may involve modulation of the regulators of the melanogenesis signal pathway instead of inhibition of intracellular tyrosinse activity.

**Table 1:** Application of the fermented broth in reducing mushroom tyrosinase activity and melanogenesis in B16 cells.

Broth	Microorganisms	Temperature	Time	Tyrosinase Activity (Mushroom)	Melanogenesis (B16 Cells)	Ref.
MRS *	*Bifidobacterium bifidum*	37 °C	48 h	↓	↓	[9]
MRS	*Bifidobacterium adolescentis*	37 °C	48 h	↓	↓	[11]
MRS	*Lactobacillus rhamnosus*	37 °C	20 h	↓	N.D. ^#^	[71]
MRS	*Bifidobacterium infantis*	37 °C	48 h	↓	↓	[79]
MRS	*Lactobacillus brevis*	37 °C	24 h	↓	N.D.	[80]
MRS	*Leuconostoc mesenteroides*	30 °C	24 h	X ^§^	↓	[46]
Soy milk	*Lactobacillus plantarum*	37 °C	48 h	↓	↓	[74]
Soybean	*Aspergillus oryzae*	-	4 months	50% ↓ 50% X	↓	[47]
Soy germ	*Aspergillus oryzae*	25 °C	1 week	↓	↓	[75]
Soybean	*Bacillus subtilis*	40 °C	36 h	↓	N.D.	[81]
Seed medium	*Streptomyces*	27 °C	96 h	X	↓	[48]
Seed medium	*Enterobacter* * sp. B20*	28 °C	5 days	↓	↓	[76]
Rice bran	*Lactobacillus rhamnosus* and Sac *charomyce cerevisiae*	15 °C	15 days	X	↓	[49]
Rice, black rice, sweet potato and barley	*Saccharomyces cerevisiae* and *Aspergillus niger*	-	-	N.D.	↓	[89]
Rice, soybean, or soygerm	*Aspergillus oryzae*	25 °C	1 week	↓	N.D.	[82]
Rice Bran papaya, and seaweed	*Lactobacillaceae*, *saccharomycetes*, *funguses*, *actinomyces* and *photosynthetic bacteria*	35 °C	7 days	↓	N.D.	[83]
*P.* *linteus* complex culture	*Phellinus linteus*	28 °C	9 days	↓	↓	[77]
Purple plain rice	Look Pang (yeasts and molds)	-	8 days	↓	N.D.	[84]
Squid pen	*Burkholderia cepacia*	30 °C	3 days	↓	N.D.	[85]
*Rhodiola rosea*	*Alcaligenes piechaudii*	30 °C	5 days	↓	N.D.	[86]
*Lonicera japonica*	*Alcaligenes piechaudii*	30 °C	5 days	↓	N.D.	[86]
*Codonopsis lanceolata*	*Bifidobacterium longum*	37 °C	7 days	↓	N.D.	[87]
*Codonopsis lanceolata*	*Lactobacillus rhamnosus*	37 °C	7 days	↓	N.D.	[87]
Potato dextrose agar	*Aspergillus oryzae*	30 °C	2 days	↓	N.D.	[88]
*Viola mandshurica*	Microorganisms	-	6 months	↓	↓	[90]

*: MRS, De Man Rogosa Sharpe; ^#^: N.D., not determined; ^§^: X, no inhibition.

### 2.2. Possible Functional Components of Fermented Broth

The components of fermented broth include carbohydrates, alcohols, alditols, glycols, and other metabolic products [73]. According to Table 1, *Lactobacilli* and *Bifidobacteria* are the two major genera of bacteria of which the broth is used to suppress melanogenesis; scientists have long used them to solve dermal problems [90]. Various studies have also used broth fermented by bacteria or fungi not classified as *Lactobacilli* and *Bifidobacteria* to inhibit melanogenesis. However, the mechanism of action of fermented broth in skin pigmentation regulation remains unclear. The possible active ingredients that could suppress melanogenesis are listed in Table 2 and discussed below.

#### 2.2.1. Lactic Acid

The lactic acid contained in *Lactobacillus rhamnosus* is crucial to inhibit tyrosinase activity and can reduce melanogenesis [71] (Table 1). Lactic acid is an effective exfoliating agent that improves skin color [91] and can suppress tyrosinase activity [92]. One report indicated that lactic acid can increase epidermal thickness, reduce melanin deposition, and upregulate collagen levels because it is derived from sour milk, like α-hydroxyacids (AHAs) [18]. AHAs accelerate desquamation of the stratum corneum to reduce tyrosinase without affecting messenger-RNA or protein expression [93]. In short, lactic acid functions as other enhancers of epidermal turnover do.

**Table 2:** Active ingredients and hypopigmentation mechanism of fermented broths.

Broth/Microorganism	Possible Active Ingredients (PAI)	Levels of PAI	Hypopigmentation Mechanism	Refs.
*P.* *linteus* complex culture/*Phellinus linteus*	Phenolics and flavonoids	↑	Inhibit tyrosinase activity, down regulate MITF, TRP1 and TRP2; activation of the phosphatidylinositol 3-kinase/Akt/glycogen synthase kinase-3beta	[77]
Soy milk/*Lactobacillus plantarum*	Aglycone isoflavones (such as dadzein and genistein)	↑	Inhibit tyrosinase activity, down regulate MITF, inactive MAPK and p38	[74]
Soybean*/Aspergillus oryzae*	8-Hydroxydaidzein and 3-hydroxydaidzein	↑	Repress MITF, decrease expression of tyrosinase, TRP1 and TRP2	[47]
Soy germ/*Aspergillus oryzae*	8-Hydroxydaidzein	↑	Inhibit tyrosinase activity	[75]
MRS*/Bifidobacterium bifidum*; MRS*/Bifidobacterium adolescentis*	Unknown	N.D.	Antioxidative activity	[9,11]
Rice, black rice, sweet potato and barley/*Saccharomyces cerevisiae* and *Aspergillus niger*	Polyphenolic compounds	barley > black rice > sweet potato > rice	Antioxidative activity	[89]
Seed edium/*Enterobacter sp. B20*	Byelyankacin	↑	Inhibit tyrosinase activity	[76]
Seed medium/*Stveptomyces* sp.	Albocycline K3	↑	Unknown	[48]
MRS*/Leuconostoc mesenteroides*	Crude self-digestion (autolysis) extract	N.D.	Inhibits tyrosinase activity, tyrosinase translation, or accelerating its degradation	[46]

N.D., not determined.

#### 2.2.2. Flavonoids

Previous research has explored fermenting plants such as ginseng, rice bran, and meshima to enhance their bioactivity and active contents [80]. Fermented rice bran is rich in kaempferol, which is a flavonoid that can reduce tyrosinase [94]. A paper discussing raw materials (nonfermented rice bran) determined that they do not inhibit tyrosinase activity [83]. Similarly, fermented red ginseng exhibits a flavonoid content nearly 15-fold greater than that of unfermented red ginseng [80]. Fungal-fermented traditional herbs such as meshima exhibit much higher mushroom tyrosinase- and melanogenesis inhibitory activity because they have a greater flavonoid and phenolic content than unfermented products do [77] (Table 2). The hypopigmentation mechanism of fermented broth from *P.* *linteus* complex culture/*Phellinus linteus* involved inhibition of tyrosinase activity, downregulation of MITF, TRP1 and TRP2, and activation of the phosphatidylinositol 3-kinase/Akt/glycogen synthase kinase-3beta [77]. The aglycones dadzein and genistein which are isoflavonoids derived from soy milk fermented by *Lactobacillus plantarum* are constituents that inhibit melanogenesis in B16F10 melanocytes by inhibiting tyrosinase expression, downregulating MITF, inactiving MAPK and p38 [74] (Table 2). Moreover, soybeans fermented with *Aspergillus oryzae,* which is a filamentous fungus, can reduce B16 cellular melanin production by 46.7% due to increased levels of 8-hydroxydaidzein and 3-hydroxydaidzein [47] (Table 2). Hydroxydaidzeins derived from isoflavones act indirectly on melanin biosynthesis, reducing microphthalmia-associated transcription factor transcription and, thus, downregulating TRP-1 and TRP-2 expression [47] (Table 2). Flavonoids such as resveratrol [20,58], aloesin [95], and fermented soybeans and their byproducts inhibit tyrosinase. However, reports have controversially indicated that not all flavonoids lighten the skin, but rather increase melanogenesis [58,96,97].

#### 2.2.3. Antioxidants

Many antioxidants exhibit depigmentation properties that interfere with the lipid peroxidation of melanocyte membranes and increase intracellular glutathione content [98]. Intracellular glutathione acts as an antioxidant that determines the expression of a melanin-based signal [99]. Hence, antioxidants may play a crucial role in melanin production regulation. The *Bifidobacteria* reported in [9,11,79] had overpowering antioxidant activity in 2,2-diphenyl-1-picrylhydrazyl scavenging capacity and 2,2'-azino-bis(3-ethylbenzthiazoline-6-sulphonic acid) radical scavenging activity assays (Table 2). Broth from rice, black rice, sweet potato and barley fermented with *Saccharomyces cerevisiae* and *Aspergillus niger* contained higher contents of polyphenolic compounds, including protocatechuic acid, catechin, caffeic acid, ferulic acid, *p*-hydroxybenzoic acid and also exhibited potent antioxidative activity which may be related to their melanogenesis reduction [89]. The content of polyphenolic compounds is in the order of barley > black rice > sweet potato > rice.

#### 2.2.4. Novel Melanogenesis Inhibitors

There are some novel melanogenesis inhibitors such as albocycline K3 (a mcrocyclic compound) and byelyankacin purified from fermented broths of seed medium/*Stveptomyces* sp. and seed edium/*Enterobacter sp. B20*, respectively [48,76] (Table 2). The hypopigmentation mechanism of albocycline K3 is still unknown and the hypopigmentation mechanism of byelyankacin may be through inhibiting tyrosinase activity [48,76] (Table 2). The results indicated that fermented broths have great potential to produce new potent natural melanogenesis inhibitors which can be applied as beneficial and safe skin whitening products. 

Autolysate of *Leuconostoc mesenteroides* isolated from kimoto induced a decrease in melanin content in B16F0 murine melanoma cells through inhibiting tyrosinase activity, tyrosinase translation, or accelerating its degradation but did not inhibit tyrosinase activity under cell-free conditions [47]. The autolysate of *L. mesenteroides* has potential use as an effective anti-melanogenic agent. However, the active ingredient responsible has still not been determined.

## 3. Future Outlook of Fermented Broth in Cosmetic Industries

Cosmetic products using probiotics such as bacteria and yeast include aftershaves, antiaging serums, face and body lotions, hydrating creams, toothpastes, sanitary napkins, tampons, shampoos, douche gels, and oral care gums [100]. However, whitening cosmetic products containing fermented broth are limited because some obstacles such as foul odors and allergens need to be overcome [100]. Nevertheless, SK-II has launched whitening and antiaging products that contain *Saccharomycopsis* fermented broth [101,102] that have been scientifically proved to be effective in whitening. Scientists can conduct further research by adding fermented broth to cosmetic products and explore the capability of fermented broth for reducing melanogenesis. In addition, cosmetic companies can use several active ingredients that target different whitening mechanisms, such as tyrosinase inhibitors, epidermal turnover enhancers, antioxidants, TRP1 inhibitors, TRP2 inhibitors and MITF inhibitors, for effective complex mixtures [13].

## 4. Conclusions

This study verifies that fermented broth can be applied in reducing melanogenesis. Active ingredient levels of fermented broth are increased compared to unfermented broths. There are lots of fermented broths still needed to be tested as melanogenesis inhibitors. In addition to inhibiting tyrosinase activity, melanogenesis inhibitors can modulate different regulators of melanogenesis via the cAMP or MAPK signal pathways. In general, optimized fermented broth culture is promising for the development of novel skin whitening ingredients.

## References

[1] Xie Q., Zhang M. (2013). White or tan? A cross-cultural analysis of skin beauty advertisements between China and the United States. Asian J. Commun..

[2] Li E.P., Min H.J., Belk R.W., Kimura J., Bahl S. (2008). Skin lightening and beauty in four asian cultures. Adv. Consum. Res..

[3] Ashikari M. (2003). Urban middle-class Japanese women and their white faces: Gender, ideology, and representation. Ethos.

[4] Cao S. (2014). Intertextuality and glocalization a corpus-based analysis of advertisement texts of an international female fashion magazine. J. Arts Hum..

[5] Li H. (2013). TCM in skin whitening and lightening: The eternal pursuit in east asia. Cosmet. Toiletries.

[6] Dixson B.J., Dixson A.F., Li B., Anderson M. (2007). Studies of human physique and sexual attractiveness: Sexual preferences of men and women in China. Am. J. Hum. Biol..

[7] Ashikari M. (2005). Cultivating Japanese whiteness: The ‘whitening’ cosmetics boom and the Japanese identity. J. Mater. Cult..

[8] Lagouvardos P.E., Tsamali I., Papadopoulou C., Polyzois G. (2013). Tooth, skin, hair and eye colour interrelationships in Greek young adults. Odontology.

[9] Huang H.C., Huang W.Y., Chiu S.H., Ke H.J., Chiu S.W., Wu S.Y., Kuo F.S., Chang T.M. (2011). Antimelanogenic and antioxidative properties of Bifidobacterium bifidum. Arch. Dermatol. Res..

[10] Kim Y.J., Uyama H. (2005). Tyrosinase inhibitors from natural and synthetic sources: Structure, inhibition mechanism and perspective for the future. Cell. Mol. Life Sci..

[11] Huang H.C., Chang T.M. (2012). Antioxidative properties and inhibitory effect of Bifidobacterium adolescentis on melanogenesis. World J. Microbiol. Biotechnol..

[12] Chang T.S. (2009). An updated review of tyrosinase inhibitors. Int. J. Mol. Sci..

[13] Smit N., Vicanova J., Pavel S. (2009). The hunt for natural skin whitening agents. Int. J. Mol. Sci..

[14] Badreshia-Bansal S., Draelos Z.D. (2007). Insight into skin lightening cosmeceuticals for women of color. J. Drugs Dermatol..

[15] Sato K., Morita M., Ichikawa C., Takahashi H., Toriyama M. (2008). Depigmenting mechanisms of all-trans retinoic acid and retinol on B16 melanoma cells. Biosci. Biotechnol. Biochem..

[16] Ando H., Itoh A., Mishima Y., Ichihashi M. (1995). Correlation between the number of melanosomes, tyrosinase mRNA levels, and tyrosinase activity in cultured murine melanoma cells in response to various melanogenesis regulatory agents. J. Cell. Physiol..

[17] Wiechers J., Rawlings A., Garcia C., Chesne C., Balaguer P., Nicolas J., Corre S., Galibert M.D. (2005). A new mechanism of action for skin whitening agents: Binding to the peroxisome proliferator-activated receptor1. Int. J. Cosmet. Sci..

[18] Ebanks J.P., Wickett R.R., Boissy R.E. (2009). Mechanisms regulating skin pigmentation: The rise and fall of complexion coloration. Int. J. Mol. Sci..

[19] Picardo M., Carrera M. (2007). New and experimental treatments of cloasma and other hypermelanoses. Dermatol. Clin..

[20] Solano F., Briganti S., Picardo M., Ghanem G. (2006). Hypopigmenting agents: An updated review on biological, chemical and clinical aspects. Pigm. Cell Res..

[21] Wood J.M., Schallreuter K.U. (1991). Studies on the reactions between human tyrosinase, superoxide anion, hydrogen peroxide and thiols. Biochim. Biophys. Acta.

[22] Shivhare S., Malviya K., Malviya K., Jain V. (2013). A review: Natural skin lighting and nourishing agents. Res. J. Top. Cosmet. Sci..

[23] Huang C.H., Sung H.C., Hsiao C.Y., Hu S., Ko Y.S. (2013). Transdermal delivery of three vitamin C derivatives by Er: YAG and carbon dioxide laser pretreatment. Lasers Med. Sci..

[24] Yao C.L., Lin Y.M., Mohamed M.S., Chen J.H. (2013). Inhibitory effect of ectoine on melanogenesis in B16-F0 and A2058 melanoma cell lines. Biochem. Eng. J..

[25] Kumar K., Vani M.G., Wang S.Y., Liao J.W., Hsu L.S., Yang H.L., Hseu Y.C. (2013). *In vitro* and *in vivo* studies disclosed the depigmenting effects of gallic acid: A novel skin lightening agent for hyperpigmentary skin diseases. Biofactors.

[26] Gonçalez M., Corrêa M., Chorilli M. (2013). Skin delivery of kojic acid-loaded nanotechnology-based drug delivery systems for the treatment of skin aging. BioMed Res. Int..

[27] Ki D.H., Jung H.C., Noh Y.W., Thanigaimalai P., Kim B.H., Shin S.C., Jung S.H., Cho C.W. (2013). Preformulation and formulation of newly synthesized QNT3-18 for development of a skin whitening agent. Drug Dev. Ind. Pharm..

[28] Won Y.-K., Loy C.-J., Randhawa M., Southall M.D. (2014). Clinical efficacy and safety of 4-hexyl-1, 3-phenylenediol for improving skin hyperpigmentation. Arch. Dermatol. Res..

[29] Son K., Heo M. (2013). The evaluation of depigmenting efficacy in the skin for the development of new whitening agents in Korea. Int. J. Cosmet. Sci..

[30] Chen Y.S., Lee S.M., Lin C.C., Liu C.Y., Wu M.C., Shi W.L. (2013). Kinetic study on the tyrosinase and melanin formation inhibitory activities of carthamus yellow isolated from *Carthamus tinctorius* L.. J. Biosci. Bioeng..

[31] Hsieh P.W., Chen W.Y., Aljuffali A., Chen C.C., Fang J.Y. (2013). Co-drug strategy for promoting skin targeting and minimizing the transdermal diffusion of hydroquinone and tranexamic acid. Curr. Med. Chem..

[32] Tse T.W., Hui E. (2013). Tranexamic acid: An important adjuvant in the treatment of melasma. J. Cosmet. Dermatol..

[33] Eimpunth S., Wanitphadeedecha R., Manuskiatti W. (2013). A focused review on acne-induced and aesthetic procedure-related postinflammatory hyperpigmentation in Asians. J. Eur. Acad. Dermatol..

[34] Amer M., Metwalli M. (1998). Topical hydroquinone in the treatment of some hyperpigmentary disorders. Int. J. Dermatol..

[35] Haddad A.L., Matos L.F., Brunstein F., Ferreira L.M., Silva A., Costa D. (2003). A clinical, prospective, randomized, double-blind trial comparing skin whitening complex with hydroquinone *vs*. placebo in the treatment of melasma. Int. J. Dermatol..

[36] Josefina N.S., Pablo C.C.J., Bertha T.Á., Cuauhtemoc O.O., Cornelia F.A., González F.J., David M.R.J., Benjamin M. (2011). A double-blind, randomized clinical trial of niacinamide 4% *versus* hydroquinone 4% in the treatment of melasma. Dermatol. Res. Pract..

[37] Spínola V., Mendes B., Câmara J.S., Castilho P.C. (2013). Effect of time and temperature on vitamin C stability in horticultural extracts. UHPLC-PDA *vs*. iodometric titration as analytical methods. LWT-Food Sci. Technol..

[38] Ookubo N., Michiue H., Kitamatsu M., Kamamura M., Nishiki T., Ohmori I., Matsui H. (2014). The transdermal inhibition of melanogenesis by a cell-membrane-permeable peptide delivery system based on poly-arginine. Biomaterials.

[39] Fujimoto N., Watanabe H., Nakatani T., Roy G., Ito A. (1998). Induction of thyroid tumours in (C57BL/6N × C3H/N) F_1_ mice by oral administration of kojic acid. Food Chem. Toxicol..

[40] Takizawa T., Mitsumori K., Tamura T., Nasu M., Ueda M., Imai T., Hirose M. (2003). Hepatocellular tumor induction in heterozygous p53-deficient CBA mice by a 26-week dietary administration of kojic acid. Toxicol. Sci..

[41] Arulmozhi V., Pandian K., Mirunalini S. (2013). Ellagic acid encapsulated chitosan nanoparticles for drug delivery system in human oral cancer cell line (KB). Colloids Surf. B.

[42] Findlay G.H., Morrison J., Simson I. (1975). Exogenous ochronosis and pigmented colloid milium from hydroquinone bleaching creams. Brit. J. Dermatol..

[43] Charlín R., Barcaui C.B., Kac B.K., Soares D.B., Rabello-Fonseca R., Azulay-Abulafia L. (2008). Hydroquinone-induced exogenous ochronosis: A report of four cases and usefulness of dermoscopy. Int. J. Dermatol..

[44] Briganti S., Camera E., Picardo M. (2003). Chemical and instrumental approaches to treat hyperpigmentation. Pigm. Cell Res..

[45] Kiken D.A., Cohen D.E. (2002). Contact dermatitis to botanical extracts. Dermatitis.

[46] Kondo S., Takahashi T., Yoshida K., Mizoguchi H. (2012). Inhibitory effects of autolysate of *Leuconostoc mesenteroides* isolated from kimoto on melanogenesis. J. Biosci. Bioeng..

[47] Goh M.J., Park J.S., Bae J.H., Kim D.H., Kim H.K., Na Y.J. (2012). Effects of ortho-dihydroxyisoflavone derivatives from Korean fermented soybean paste on melanogenesis in B16 melanoma cells and human skin equivalents. Phytother. Res..

[48] Takamatsu S., Kim Y., Hayashi M., Komiyama K., Imokawa G., Omura S. (1995). A new inhibitor of melanogenesis, albocycline K3, produced by *Stveptomyces sp.* OH-3984. Tetrahedron Lett..

[49] Chung S.Y., Seo Y.K., Park J.M., Seo M.J., Park J.K., Kim J.W., Park C.S. (2009). Fermented rice bran bownregulates MITF expression and leads to inhibition of α-MSH-induced melanogenesis in B16F1 melanoma. Biosci. Biotechnol. Biochem..

[50] Bae-Harboe Y.S., Park H.Y. (2012). Tyrosinase: A central regulatory protein for cutaneous pigmentation. J. Investig. Dermatol..

[51] Tepper A.W.J.W. (2005). Tyrosinase: Biology, structure and mechanism. Structure and Mechanism of the Type-3 Copper Protein Tyrosinase.

[52] Burchill S.A., Bennett D.C., Holmes A., Thody A.J. (1991). Tyrosinase expression and melanogenesis in melanotic and amelanotic B16 mouse melanoma cells. Pathobiology.

[53] Videira I.F., Moura D.F., Magina S. (2013). Mechanisms regulating melanogenesis. An. Bras. Dermatol..

[54] Park H.Y., Kosmadaki M., Yaar M., Gilchrest B.A. (2009). Cellular mechanisms regulating human melanogenesis. Cell. Mol. Life Sci..

[55] Lee J.Y., Choi H.J., Chung T.W., Kim C.H., Jeong H.S., Ha K.T. (2013). Caffeic acid phenethyl ester inhibits alpha-melanocyte stimulating hormone-induced melanin synthesis through suppressing transactivation activity of microphthalmia-associated transcription factor. J. Nat. Prod..

[56] Wu L.C., Lin Y.Y., Yang S.Y., Weng Y.T., Tsai Y.T. (2011). Antimelanogenic effect of c-phycocyanin through modulation of tyrosinase expression by upregulation of ERK and downregulation of p38 MAPK signaling pathways. J. Biomed. Sci..

[57] Saha B., Singh S.K., Sarkar C., Bera R., Ratha J., Tobin D.J., Bhadra R. (2006). Activation of the Mitf promoter by lipid-stimulated activation of p38-stress signalling to CREB. Pigm. Cell Res..

[58] Lin C., Babiarz L., Liebel F., Price E.R., Kizoulis M., Gendimenico G., Fisher D., Seiberg M. (2002). Modulation of microphthalmia-associated transcription factor gene expression alters skin pigmentation. J. Investig. Dermatol..

[59] Jiang Z., Li S., Liu Y., Deng P., Huang J., He G. (2011). Sesamin induces melanogenesis by microphthalmia-associated transcription factor and tyrosinase up-regulation via cAMP signaling pathway. Acta Biochim. Biophys. Sin..

[60] Lee C.S., Jang W.H., Park M., Jung K., Baek H.S., Joo Y.H., Park Y.H., Lim K.M. (2013). A novel adamantyl benzylbenzamide derivative, AP736, suppresses melanogenesis through the inhibition of cAMP-PKA-CREB-activated microphthalmia-associated transcription factor and tyrosinase expression. Exp. Dermatol..

[61] Shibahara S., Takeda K., Yasumoto K., Udono T., Watanabe K., Saito H., Takahashi K. (2001). Microphthalmia-associated transcription factor (MITF): Multiplicity in structure, function, and regulation. J. Investig. Dermatol. Symp. Proc..

[62] Huang H.C., Chang S.J., Wu C.Y., Ke H.J., Chang T.M. (2014). [6]-Shogaol inhibits alpha-MSH-induced melanogenesis through the acceleration of ERK and PI3K/Akt-mediated MITF degradation. Biomed. Res. Int..

[63] Su T.R., Lin J.J., Tsai C.C., Huang T.K., Yang Z.Y., Wu M.O., Zheng Y.Q., Su C.C., Wu Y.J. (2013). Inhibition of melanogenesis by gallic acid: Possible involvement of the PI3K/Akt, MEK/ERK and Wnt/beta-catenin signaling pathways in B16F10 cells. Int. J. Mol. Sci..

[64] Dong Y., Wang H., Cao J., Ren J., Fan R., He X., Smith G.W., Dong C. (2011). Nitric oxide enhances melanogenesis of alpaca skin melanocytes *in vitro* by activating the MITF phosphorylation. Mol. Cell. Biochem..

[65] Jang J.Y., Lee J.H., Jeong S.Y., Chung K.T., Choi Y.H., Choi B.T. (2009). Partially purified *Curcuma longa* inhibits alpha-melanocyte-stimulating hormone-stimulated melanogenesis through extracellular signal-regulated kinase or Akt activation-mediated signalling in B16F10 cells. Exp. Dermatol..

[66] Ko H.H., Chiang Y.C., Tsai M.H., Liang C.J., Hsu L.F., Li S.Y., Wang M.C., Yen F.L., Lee C.W. (2014). Eupafolin, a skin whitening flavonoid isolated from Phyla nodiflora, downregulated melanogenesis: Role of MAPK and Akt pathways. J. Ethnopharmacol..

[67] Klinke H.B., Thomsen A., Ahring B.K. (2004). Inhibition of ethanol-producing yeast and bacteria by degradation products produced during pre-treatment of biomass. Appl. Microbiol. Biotechnol..

[68] Giraffa G., Chanishvili N., Widyastuti Y. (2010). Importance of lactobacilli in food and feed biotechnology. Res. Microbiol..

[69] Ouwehand A., Båtsman A., Salminen S. (2003). Probiotics for the skin: A new area of potential application?. Lett. Appl. Microbiol..

[70] Mak A.K.Y. (2007). Advertising whiteness: An assessment of skin color preferences among urban Chinese. Vis. Commun. Q..

[71] Tsai C.C., Chan C.F., Huang W.Y., Lin J.S., Chan P., Liu H.Y., Lin Y.S. (2013). Applications of Lactobacillus rhamnosus spent culture supernatant in cosmetic antioxidation, whitening and moisture retention applications. Molecules.

[72] Gaden E.L. (2000). Fermentation process kinetics. Biotechnol. Bioeng..

[73] Hanko V.P., Rohrer J.S. (2000). Determination of carbohydrates, sugar alcohols, and glycols in cell cultures and fermentation broths using high-performance anion-exchange chromatography with pulsed amperometric detection. Anal. Biochem..

[74] Chen Y.M., Shih T.W., Chiu C.P., Pan T.M., Tsai T.Y. (2013). Effects of lactic acid bacteria-fermented soy milk on melanogenesis in B16F0 melanocytes. J. Funct. Foods.

[75] Tai S.S., Lin C.G., Wu M.H., Chang T.S. (2009). Evaluation of depigmenting activity by 8-hydroxydaidzein in mouse B16 melanoma cells and human volunteers. Int. J. Mol. Sci..

[76] Takahashi S., Iwai H., Kosaka K., Miyazaki T., Osanai Y., Arao N., Tanaka K., Nagai K., Nakagawa A. (2007). Byelyankacin: A novel melanogenesis inhibitor produced by *Enterobacter sp.* B20. J. Antibiot..

[77] Cha J.Y., Yang H.J., Moon H.I., Cho Y.S. (2012). Inhibitory effect and mechanism on melanogenesis from fermented herbal composition for medical or food uses. Food Res. Int..

[78] Kwak Y.J., Kim K.S., Kim K.M., Yu H.Y., Chung E., Kim S.J., Cha J.Y., Lee Y.C., Lee J.H. (2011). Fermented *Viola mandshurica* inhibits melanogenesis in B16 melanoma cells. Biosci. Biotechnol. Biochem..

[79] Huang H.C., Chiu S.H., Ke H.J., Chiu S.W., Wu S.Y., Chang T.M. (2011). Antimelanogenic and antioxidant activities of Bifidobacterium infantis. Afr. J. Microbiol. Res..

[80] Lee H.S., Kim M.R., Park Y., Park H.J., Chang U.J., Kim S.Y., Suh H.J. (2012). Fermenting red ginseng enhances its safety and efficacy as a novel skin care anti-aging ingredient: *In vitro* and animal study. J. Med. Food.

[81] Chae G.Y., Ha B.J. (2011). The comparative evaluation of fermented and non-fermented soybean extract on antioxidation and whitening. Toxicol. Res..

[82] Chang T.S., Ding H.Y., Tai S.S.K., Wu C.Y. (2007). Mushroom tyrosinase inhibitory effects of isoflavones isolated from soygerm koji fermented with Aspergillus oryzae BCRC 32288. Food Chem..

[83] Li Z., Lee J., Cho M.H. (2010). Antioxidant, antibacterial, tyrosinase inhibitory, and biofilm inhibitory activities of fermented rice bran broth with effective microorganisms. Biotechnol. Bioprocess Eng..

[84] Manosroi A., Ruksiriwanich W., Manosroi J. (2008). Free radical scavenging and tyrosinase inhibition activities of fermented Thai rice for cosmeceuticals. J. Thai. Tradit. Altern. Med..

[85] Hsu C.H., Nguyen A.D., Chen Y.W., Wang S.L. (2014). Tyrosinase inhibitors and insecticidal materials produced by *Burkholderia cepacia* using squid pen as the sole carbon and nitrogen source. Res. Chem. Intermed..

[86] Chen Y.S., Liou H.C., Chan C.F. (2013). Tyrosinase inhibitory effect and antioxidative activities of fermented and ethanol extracts of *Rhodiola rosea* and *Lonicera japonica*. Sci. World J..

[87] He X., Zou Y., Yoon W.B., Park S.J., Park D.S., Ahn J. (2011). Effects of probiotic fermentation on the enhancement of biological and pharmacological activities of Codonopsis lanceolata extracted by high pressure treatment. J. Biosci. Bioeng..

[88] Chang T.S., Lin M.Y., Lin H.J. (2010). Identifying 8-hydroxynaringenin as a suicide substrate of mushroom tyrosinase. J. Cosmet. Sci..

[89] Ohgidani M., Komizu Y., Goto K., Ueoka R. (2012). Antimelanogenic and antioxidative effects of residual powders from Shochu distillation remnants. Food Chem..

[90] Lew L.C., Gan C.Y., Liong M.T. (2012). Dermal bioactives from lactobacilli and bifidobacteria. Ann. Microbiol..

[91] Hasegawa S., Azuma M., Takahashi K. (2008). Stabilization of enzyme activity during the esterification of lactic acid in hydrophobic ethers and ketones as reaction media that are miscible with lactic acid despite their high hydrophobicity. Enzym. Microb. Technol..

[92] Usuki A., Ohashi A., Sato H., Ochiai Y., Ichihashi M., Funasaka Y. (2003). The inhibitory effect of glycolic acid and lactic acid on melanin synthesis in melanoma cells. Exp. Dermatol..

[93] Bowe W.P., Shalita A.R. (2008). Effective over-the-counter acne treatments. Semin. Cutan. Med. Surg..

[94] Lim Y.H., Kim I.H., Seo J.J., Kim J.K. (2006). Tyrosinase inhibitor from the flowers of Impatiens balsamina. J. Microbiol. Biotechnol..

[95] Ken J., Jennifer H., Mei H., Qi J., Steve O. (2002). Modulation of melanogenesis by aloesin: A competitive inhibitor of tyrosinase. Pigm. Cell Res..

[96] Fu B., Li H., Wang X., Lee F.S., Cui S. (2005). Isolation and identification of flavonoids in licorice and a study of their inhibitory effects on tyrosinase. J. Agric. Food Chem..

[97] Nerya O., Vaya J., Musa R., Izrael S., Ben-Arie R., Tamir S. (2003). Glabrene and isoliquiritigenin as tyrosinase inhibitors from licorice roots. J. Agric. Food Chem..

[98] Del Marmol V., Solano F., Sels A., Huez G., Libert A., Lejeune F., Ghanem G. (1993). Glutathione depletion increases tyrosinase activity in human melanoma cells. J. Investig. Dermatol..

[99] Galván I., Alonso-Alvarez C. (2008). An intracellular antioxidant determines the expression of a melanin-based signal in a bird. PLoS One.

[100] Foligne B., Daniel C., Pot B. (2013). Probiotics from research to market: the possibilities, risks and challenges. Curr. Opin. Microbiol..

[101] Tsai H.H., Chen Y.C., Lee W.R., Hu C.H., Hakozaki T., Yoshii T., Shen S.C. (2006). Inhibition of inflammatory nitric oxide production and epidermis damages by *Saccharomycopsis* ferment filtrate. J. Dermatol. Sci..

[102] Pang J.H., Wong W.R., Hakozaki T., Yoshii T., Chen T.Y. (2011). Up-regulation of tight junction-related proteins and increase of human epidermal keratinocytes barrier function by *Saccharomycosis* ferment filtrate. J. Cosmet. Dermatol. Sci. Appl..

